# Causal relationship between bone mineral density and intervertebral disc degeneration: a univariate and multivariable mendelian randomization study

**DOI:** 10.1186/s12891-024-07631-7

**Published:** 2024-07-05

**Authors:** Luming Li, Dawei Li, Ziming Geng, Zhenxin Huo, Yuxiang Kang, Xiangxiang Guo, Bing Yuan, Baoshan Xu, Tao Wang

**Affiliations:** 1 Tianjin Medical University, NO. 22, Qi Xiang Tai Road, Heping District, Tianjin, 300070 China; 2 Department of Minimally Invasive Spine Surgery, Tianjin Hospital, No. 406, Jie Fang Nan Road, Hexi District, Tianjin, 300211 China; 3 Tianjin TEDA Hospital, No. 61, Third Street, Binhai New Area, Tianjin, 300457 China; 4 Department of Spine Surgery, Tianjin Hospital, Tianjin University, No. 406 Jiefang South Rd, Hexi District, Tianjin, 300211 China; 5 The Fifth Hospital of Wuhan, The Second Affiliated Hospital of Jianghan University, No. 122 Xianzheng Street, Hanyang District, Wuhan, Hubei 430050 China

**Keywords:** Osteoporosis, Bone mineral density, Intervertebral disc degeneration, Mendelian randomization (MR), GWAS data

## Abstract

**Background:**

Although previous studies have suggested a possible association between bone mineral density (BMD) and intervertebral disc degeneration (IDD), the causal relationship between them remains unclear. Evidence from accumulating studies indicates that they might mutually influence one another. However, observational studies may be affected by potential confounders. Meanwhile, Mendelian randomization (MR) study can overcome these confounders to assess causality.

**Objectives:**

This Mendelian randomization (MR) study aimed to explore the causal effect of bone mineral density (BMD) on intervertebral disc degeneration (IDD).

**Methods:**

Summary data from genome-wide association studies of bone mineral density (BMD) and IDD (the FinnGen biobank) have been acquired. The inverse variance weighted (IVW) method was utilized as the primary MR analysis approach. Weighted median, MR-Egger regression, weighted mode, and simple mode were used as supplements. The Mendelian randomization pleiotropy residual sum and outlier (MR-PRESSO) and MR-Egger regression were performed to assess horizontal pleiotropy. Cochran’s Q test evaluated heterogeneity. Leave-one-out sensitivity analysis was further conducted to determine the reliability of the causal relationship. Multivariate MR (MVMR) analyses used multivariable inverse variance-weighted methods to individually and jointly adjust for four potential confounders, body mass index (BMI), Type2 diabetes, hyperthyroidism and smoking. A reverse MR analysis was conducted to assess potential reverse causation.

**Results:**

In the univariate MR analysis, femoral neck bone mineral density (FNBMD), heel bone mineral density (eBMD), lumbar spine bone mineral density (LSBMD), and total body bone mineral density (TB BMD) had a direct causal effect on intervertebral disc degeneration (IDD) [FNBMD-related analysis: OR(95%CI) = 1.17 (1.04 to 1.31), *p* = 0.008, eBMD-related analysis: OR(95%CI) = 1.06 (1.01 to 1.12), *p* = 0.028, LSBMD-related analysis: OR(95%CI) = 1.20 (1.10 to 1.31), *p* = 3.38E-7,TB BMD-related analysis: OR(95%CI) = 1.20 (1.12 to 1.29), *p* = 1.0E-8]. In the MVMR analysis, it was revealed that, even after controlling for confounding factors, heel bone mineral density (eBMD), lumbar spine bone mineral density (LSBMD), and total body bone mineral density (TB BMD) still maintained an independent and significant causal association with IDD(Adjusting for heel bone mineral density: beta = 0.073, OR95% CI = 1.08(1.02 to 1.14), *P* = 0.013; Adjusting for lumbar spine bone mineral density: beta = 0.11, OR(95%CI) = 1.12(1.02 to 1.23), *P* = 0.03; Adjusting for total body bone mineral density: beta = 0.139, OR95% CI = 1.15(1.06 to 1.24), *P* = 5.53E − 5). In the reverse analysis, no evidence was found to suggest that IDD has an impact on BMD.

**Conclusions:**

The findings from our univariate and multivariable Mendelian randomization analysis establish a substantial positive causal association between BMD and IDD, indicating that higher bone mineral density may be a significant risk factor for intervertebral disc degeneration. Notably, no causal effect of IDD on these four measures of bone mineral density was observed. Further research is required to elucidate the underlying mechanisms governing this causal relationship.

**Supplementary Information:**

The online version contains supplementary material available at 10.1186/s12891-024-07631-7.

## Introduction

Intervertebral disc degeneration (IDD) is one of the leading causes of chronic low back pain. The degenerative cascade is often initiated by an imbalance between catabolic and anabolic processes in the intervertebral discs [1]. As a consequence of extracellular matrix degradation, neoinnervation and neovascularization take place. Ultimately, this degenerative process results in disc bulging and loss of nucleus pulposus which eventually lead to conditions such as disc herniations, spinal stenosis, and degenerative spondylolisthesis, all of which can give rise to low back pain and, in severe cases, lead to complete incapacity and disability. To date, in spite of the high prevalence of IDD, lines of evidence for the risk factors of IDD have not been fully established yet. It may be the result of a combination of genetic background and environmental factors including aging, obesity, physical activity, and smoking [2, 3].

Osteoporosis (OP) and intervertebral disc degeneration (IDD) are prevalent conditions among the elderly population [4, 5].Both significantly diminish quality of life and pose substantial economic burdens on society. These geriatric chronic diseases, osteoporosis (OP) and intervertebral disc degeneration (IDD), commonly coexist within the same elderly individuals due to their high incidence rates. It has been reported that patients with osteoporosis have more severe disc degeneration [6]. However, numerous studies have indicated a positive association between lumbar disc degeneration and bone mineral density (BMD), signifying that higher vertebral bone density is linked to lumbar disc degeneration [7–14]. One potential explanation for this association may be that the presence of osteophytes and lumbar spine fractures could potentially lead to an overestimation of bone mineral density measurementsStyrkarsdottir, Halldorsson [15]. The contradictory findings in existing studies pose a challenge in establishing a causal relationship between osteoporosis (OP) and intervertebral disc degeneration (IDD), especially in the presence of unmeasured confounding factors like age, obesity, and smoking, which can contribute to both OP and IDD. Investigating the causal link between bone mineral density (BMD) and IDD can provide valuable insights into novel approaches for preventing, diagnosing, and managing IDD.

Mendelian randomization (MR) studies, which use an epidemiological approach that assesses the causal effect of a risk factor on an outcome, have been increasingly used to overcome the aforementioned limitations and explore causal relationships [16]. At present, bidirectional association studies on the association between BMD and IDD are rare, and longitudinal investigations of the relationship have produced inconsistent results. Multivariate Mendelian randomization (MVMR) is an expansion of MR that takes into account pleiotropy amongst multiple traits, allowing multiple potentially highly relevant exposures to be included in MR estimates and estimates the direct causal impact of one or more exposures of interest on outcomes. [17]

Hence, in this study, we gathered published data on four types of BMD and IDD from a comprehensive GWAS. And we conducted Mendelian randomization (MR) analyses utilizing both univariate and multivariate bidirectional approaches to assess the causal relationship between bone mineral density (BMD) and the risk of intervertebral disc degeneration (IDD), as well as the causal impact of BMD on IDD.

## Materials and methods

### Study design

**Fig. 1:**
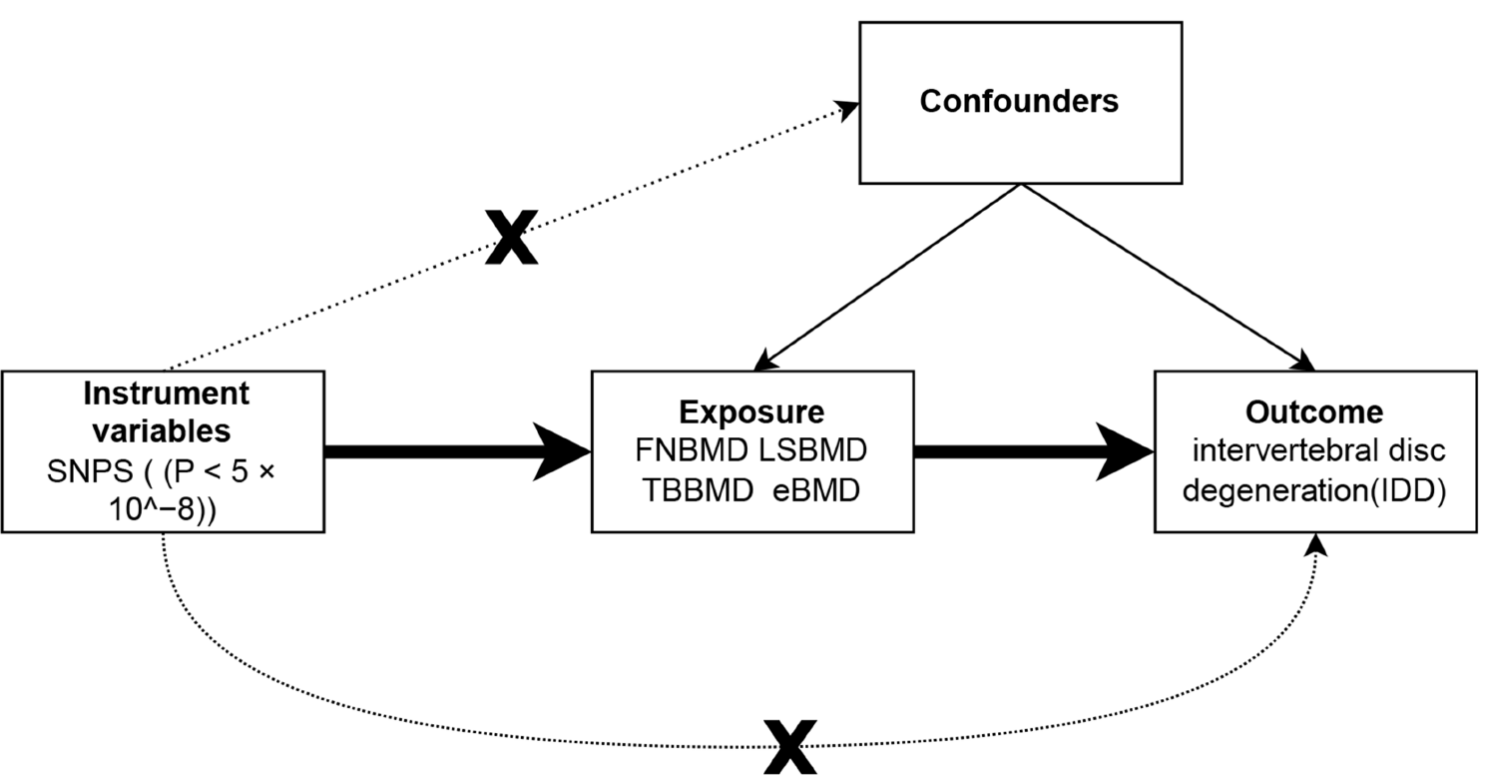
Overall flow chart of the univariable MR study design

**Fig. 2:**
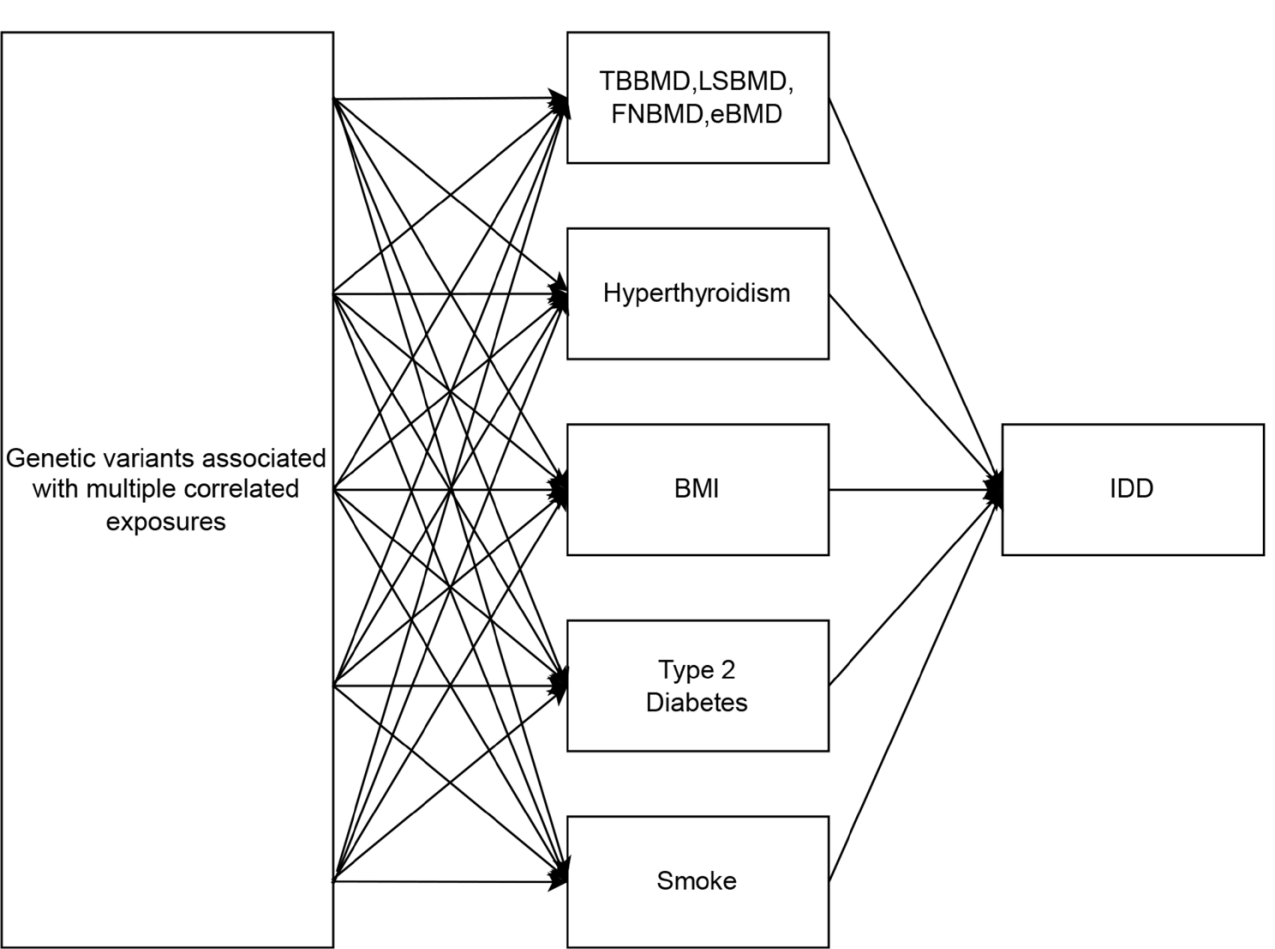
Overview of the multivariable MR study design

The overall flowchart of this study is shown in Fig. 1. MR studies require three assumptions to be met: [1] a strong correlation between the instrumental variable (IV) and the exposure, [2] IVs are unrelated to confounding factors, and [3] IVs are only related to the outcome through the exposure [18, 19]. Specifically, we determined the bone mineral density that had a causal effect on intervertebral disc degeneration (IDD) through bidirectional two-sample Mendelian randomization. Previous studies have provided evidence of genetic associations between multiple traits and BMD and IDD, including Type 2 diabetes [3],body mass index (BMI) [20],Hyperthyroidism [21] and smoking. Therefore, these four variables were individually subjected to multivariable MR (MVMR) with four types of bone density (Fig. 2). All data for this study were obtained from the publicly available GWAS study database and therefore did not require ethical approval.

### Data sources for exposure and outcome

Clinically, femoral neck bone mineral density (FN BMD), lumbar spine bone mineral density (LS BMD), Total body bone mineral density(TB BMD)and Heel bone mineral density (eBMD) have been widely used as measurable and powerful predictors of osteoporosis [17]. BMD is highly heritable and associated with common genetic variants [15]. We extracted instrumental variables for four types of BMD (bone mineral density) from the IEU GWAS database (https://gwas.mrcieu.ac.uk/datasets/). femoral neck bone mineral density (FN BMD) was derived from a GWAS involving 32,735 individuals (ID: ieu-a-980), lumbar spine bone mineral density (LS BMD) from a GWAS involving 28,498 individuals (ID: ieu-a-982), Total body bone mineral density (TB BMD) from a GWAS involving 56,284 individuals (ID: ebi-a-GCST005348), and Heel bone mineral density (e BMD) from a GWAS involving 426,824 individuals (ID: ebi-a-GCST006979). The genetic values of the individuals were corrected for sex, age, and weight, and then standardized. Relevant ethics committees approved all studies contributing data to these analyses.

We sourced summary statistics for the Genome-Wide Association Study (GWAS) on Intervertebral Disc Degeneration (IDD) from the FinnGen Consortium release, encompassing a vast dataset of 29,508 cases and 227,388 controls [22]. The diagnosis of Intervertebral Disc Degeneration (IDD) was based on standardized coding systems, specifically ICD-10 M51, ICD-9 722, and ICD-8 275, with specific exclusions made for certain codes (ICD-9 7220|7224|7227|7228 A, ICD-8 7250). For a comprehensive overview of the exposure and outcome variables analyzed in this Mendelian Randomization (MR) study, please refer to Table 1, which provides detailed information.

**Table 1:** The details of the exposure and outcome. GEFOS, Genetic Factors for Osteoporosis (GEFOS) Consortium

Trait	Consortium	Samples	Cases	Control	Population
**exposure**					
FNBMD	GEFOS	32,735	/	/	Mixed
TBBMD	GEFOS	56,284	/	/	European
eBMD	UK Biobank	426,824	/	/	European
LSBMD	GEFOS	28,498			Mixed
**Outcome**					
Intervertebral disc degeneration	FinnGen R9	256,896	29,508	227,388	European

### Genetic instrumental variable selection

In accordance with the three assumptions for MR analysis, independent single-nucleotide polymorphisms (SNPs) associated with the exposure at the genome-wide significance level (*p* < 5 × 10⁻⁸) were selected as instrumental SNPs (clumping r² = 0.001 and kb = 10,000) [23]. Subsequently, we eliminated palindromic SNPs and SNPs that did not appear in the outcome from the IVs. The association values of the corresponding SNPs were also obtained from outcome GWAS summary statistics. After harmonizing, we calculated the F statistic to evaluate the strength of selected IVs. Genetic IV with F statistics > 10 indicated a good strength of instrument to alleviate potential bias in MR analysis. R² represents the proportion of variance in the exposure that is explained by genetic variants. The calculation formula is as follows: $\varvec{F}=\frac{ \mathbf{N}-\mathbf{K}-1 }{\mathbf{K} }\times \frac{\varvec{R}^2}{1-\varvec{R}^2}$; $\varvec{R}^2=2\times \mathbf{E}\mathbf{A}\mathbf{F}\times (1-\mathbf{E}\mathbf{A}\mathbf{F}) \times \mathbf{B}\mathbf{E}\mathbf{T}\mathbf{A}^2$. N=sample size; K=the number of IV. If the F statistic>10, weak IVs were deemed not to have caused bias [24].

### MR analysis

The inverse variance weighted (IVW) method was employed to assess the bidirectional univariate and multivariate relationship between BMD and IDD as the primary statistical approach. The IVW method was deemed the most accurate for estimating the causal relationship, assuming no definitive evidence indicating the existence of directional pleiotropy (p for MR-Egger intercept > 0.05) [24]. When there was insufficient evidence of heterogeneity (p for MR heterogeneity > 0.05) in these selected IVs, the IVW fixed-effect model was conducted; otherwise, the IVW random-effects model was assumed [25]. A weighted median method was also conducted, which can generate effective causal estimates when at least 50% valid IVs were present in all the selected IVs [26]. All statistical analyses were performed using the “TwoSampleMR” package and the “MRPRESSO” package in R language (version 4.3.0).

### Sensitivity analysis

To test the robustness of our results, several sensitivity analyses were performed. Heterogeneity across IVs was evaluated by Cochran’s Q statistic. MR pleiotropy test was employed to perform MR Egger and returns intercept values to assess horizontal pleiotropy. MRPRESSO global test was also utilized to achieve the same objective, which eliminated the influence of pleiotropy by removing outliers [27].

## Results

### Genetic instrumental variable selection

In the univariate MR analysis, we individually filtered 19,479, 21, and 79 SNPs corresponding to FNBMD, eBMD, LSBMD, and TBBMD, respectively (Supplementary Material 1). The F-statistics for all instrumental variables were calculated and are provided in Supplementary Material Table 1. Importantly, all values surpassed 10, suggesting that the chosen IVs exhibited significant strength in alleviating potential bias.

### Causal relationship between BMD and IDD in univariable MR

**Fig. 3:**
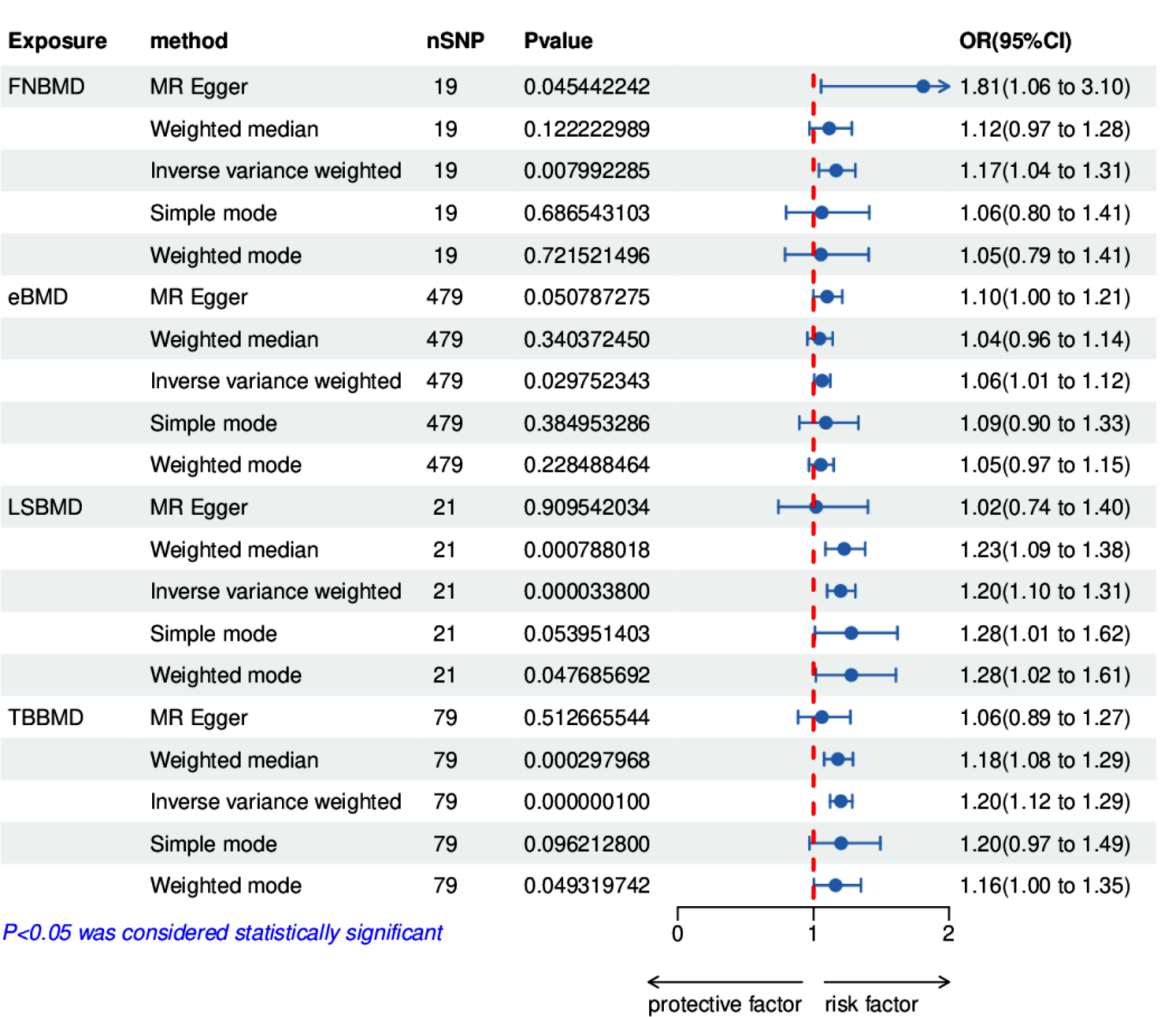
Univariable Mendelian randomization (MR) results exhibit diverse associations with intervertebral disc degeneration (IDD) across the four types of bone mineral density (BMD). BMI, body mass index (BMI); FNBMD, femoral neck bone mineral density; eBMD, Heel bone mineral density; LSBMD, lumbar spine bone mineral density; TBBMD, Total body bone mineral density

Causal Associations from BMD to IDD were identified through Univariable Mendelian randomization analysis evaluating the causal link between four kinds of BMD and IDD. As shown in Fig. 3, IVW analysis results reveals a significant association between BMD and an elevated risk of intervertebral disc degeneration [FNBMD-related analysis: 1.17 (1.04, 1.31), *p* = 0.008, eBMD-related analysis: 1.06 (1.01, 1.12), *p* = 0.028, LSBMD-related analysis: 1.20 (1.10, 1.31), *p* = 3.38E-7, TB BMD-related analysis: odds ratio (OR) = 1.20, 95% confidence interval (CI) = (1.12,1.29), *p* = 1.0E-8]. In addition, we performed four supplementary analysis methods to complement the IVW analysis, and the results were consistent with the direction of the IVW method (Fig. 3). This indicates the robustness of the IVW results.

### Multivariable MR

In addition, we performed a Multivariable Mendelian Randomization (MVMR) analysis, incorporating adjustments for Type 2 diabetes, BMI, hyperthyroidism and smoking. This was undertaken to enhance the precision of evaluating the causal relationship between genetically predicted BMD and IDD. The instrumental variables selected for MVMR implementation are presented in Supplementary Material 2.

**Fig. 4:**
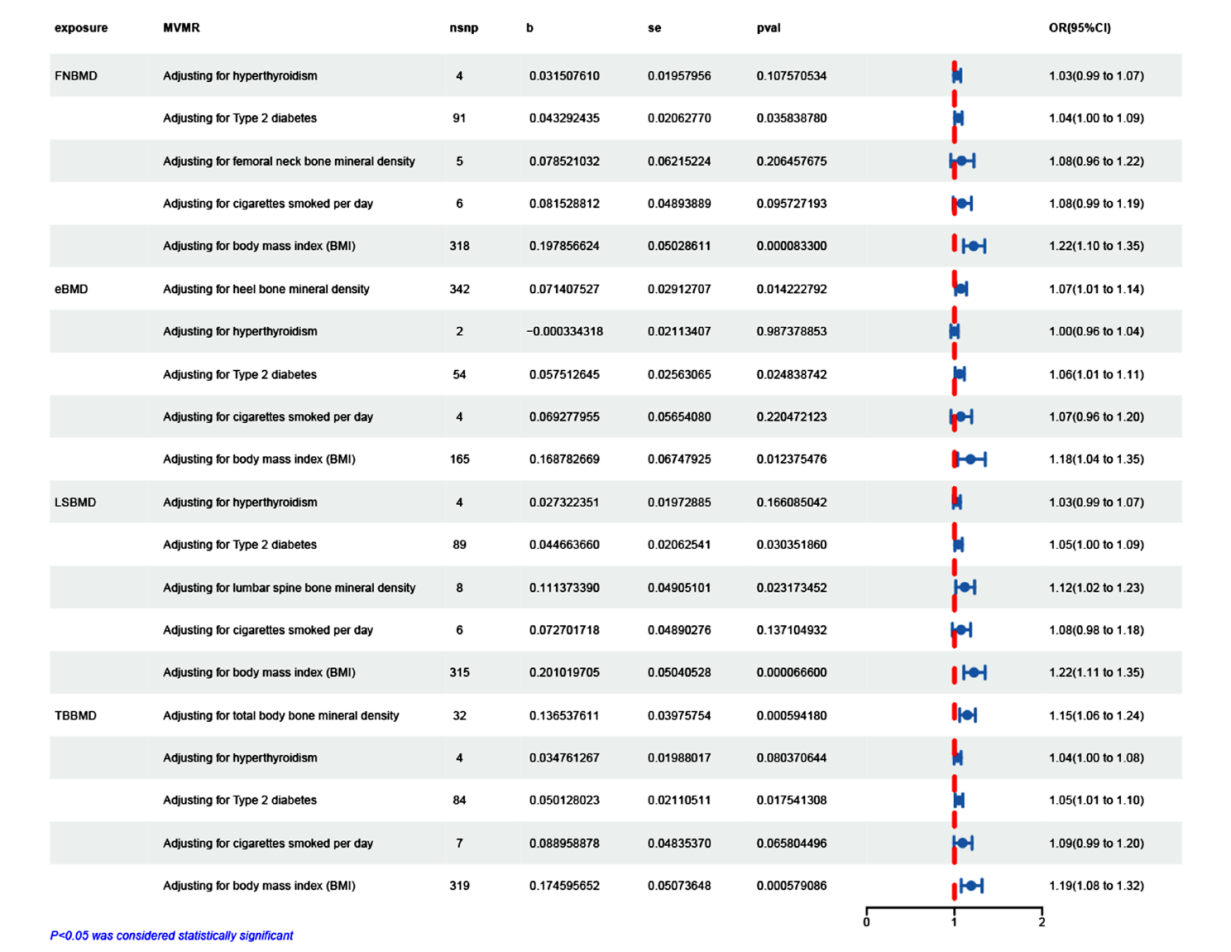
Forest plots of multivariate Mendelian randomization analyses examined causal associations between BMD and IDD, adjusted for, type 2 diabetes, BMI, smoking, and hyperthyroidism. Estimates of causality were expressed as odds ratios (ORs) and 95% confidence intervals (CIs). FNBMD, femoral neck bone mineral density; eBMD, Heel bone mineral density; LSBMD, lumbar spine bone mineral density; TBBMD, Total body bone mineral density

Multivariate IVW MR methods were used to estimate causality. MVMR analysis adjusting Type 2 diabetes, BMI Hyperthyroidism and smoking alone revealed that eBMD, LSBMD and TBBMD still had a direct causal influence on the IDD in the study of the direction of the causative effect of BMD on IDD (Adjusting for heel bone mineral density: beta = 0.073, OR95% CI = 1.08(1.02 to 1.14), *P* = 0.013; Adjusting for lumbar spine bone mineral density: beta = 0.11, OR(95%CI) = 1.12(1.02 to 1.23), *P* = 0.03; Adjusting for total body bone mineral density: beta = 0.139, OR95% CI = 1.15(1.06 to 1.24), *P* = 5.53E − 5). Strong evidence demonstrated that genetically predicted eBMD, LSBMD and TBBMD had a causal effect on IDD (Fig. 4). However, MVMR analysis adjusting Type 2 diabetes, BMI and smoking alone revealed that FNBMD didn’t have a direct causal effect on the risk of IDD in the examination of the relationship between BMD and IDD (Adjusting for femoral neck bone mineral density: beta = 0.0798, 1.08(0.96 to 1.23), *P* = 0.21) (Fig. 4).

### Sensitivity analysis and reverse analysis

**Fig. 5:**
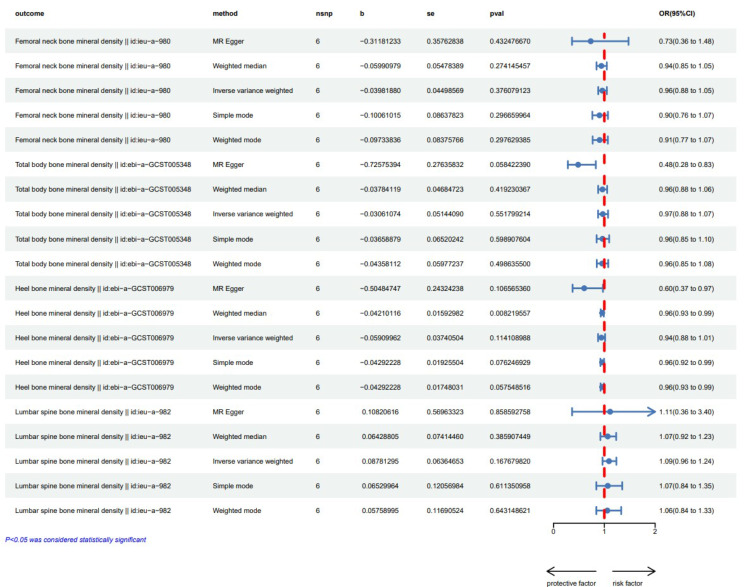
Reverse Mendelian randomization (MR) results

The MR pleiotropy test showed no horizontal pleiotropy and no outlier IV was identified in the MR-PRESSO analysis. While heterogeneity was detected by Cochran’s Q test when TBBMD and eBMD were used as exposures (Table 2), we applied a random-effects IVW method to analyze the results. The leave-one-out sensitivity analysis demonstrated that the IVW analysis results remained consistent when any SNP was removed as an instrumental variable (Supplementary Material 4). Additionally, in the reverse Mendelian randomization (MR) analysis, the results indicate that the inverse variance-weighted (IVW) values for all four bone densities are greater than 0.05. Therefore, we did not observe any significant causal effect of IDD on BMD (Fig. 5), and as a result, we did not perform MR pleiotropy test.

**Table 2:** Results of heterogeneity and horizontal pleiotropy analysis for instrumental variables (IVs) associated with the three types of bone mineral density (BMD) in relation to intervertebral disc degeneration (IDD)

Exposures	Outcomes	No. of IVs	Heterogeneity test	Pleiotropy test (MR Egger)	MRpresso
			Cochran’s Q (P valve)	Intercept (P valve)	RSS obs(P valve)
FNBMD	Intervertebral disc degeneration	19	22.77(0.16)	-0.028 (0.12)	30.61(0.068)
eBMD	Intervertebral disc degeneration	479	768.65 (<0.01)	-0.001(0.39)	805(0.902)
LSBMD	Intervertebral disc degeneration	21	19.61(0.42)	0.011 (0.31)	31.34(0.191)
TBBMD	Intervertebral disc degeneration	79	110.27(<0.01)	0.007(0.153)	124.43 (0.072)

## Discussion

The association between bone mineral density (BMD) and intervertebral disc degeneration (IDD) has garnered significant attention to date. However, the causal relationship between these two factors remains undisclosed. Drawing upon our findings, we demonstrated that BMD was an important risk factor causally associated with IDD by using univariate and multivariate MR analysis. Furthermore, we have identified a significant positive causal effect of femoral neck bone mineral density (FNBMD), heel bone mineral density (eBMD), lumbar spine bone mineral density (LSBMD), and total body bone mineral density (TB BMD) on IDD. This study has provided evidence that higher BMD is a significant risk factor causally associated with intervertebral disc degeneration, as determined through Mendelian randomization (MR) analysis. To the best of our knowledge, this is the first bi-directional univariate and multivariate Mendelian Randomization (MR) study examining the causal relationship between Bone Mineral Density (BMD) and Intervertebral Disc Degeneration (IDD) while accounting for potential confounding factors.

Our findings align with several previous studies that have suggested a potential association between higher BMD and IDD. In a prospective study conducted by Salo et al. [28]. in 2014, which involved 168 postmenopausal female patients, an investigation was undertaken to explore the relationship between lumbar disc degeneration (LDD) and the bone mineral density (BMD) of the lumbar spine and femoral neck. They discovered a correlation between higher bone mineral density and more severe intervertebral disc degeneration. Additionally, a single-center study involving 359 patients found that after controlling for age, there was a positive correlation trend between the T-score and the severity of lumbar intervertebral disc degeneration. Furthermore, they observed that lumbar disc spaces were more likely to narrow in individuals with higher bone mineral density (BMD) [8].

Considering the consistent findings of the aforementioned studies regarding the correlation between lumbar bone mineral density and intervertebral disc degeneration, a study by Miyakoshi et al. [12]. found a positive correlation between distal bone mineral density and intervertebral disc degeneration (*P* < 0.05). This suggests that higher whole-body bone mineral density may be a risk factor for intervertebral disc degeneration. Nanjo Y conducted a study with the aim of investigating the correlation between intervertebral disk degeneration and bone mineral density. Results showed that in premenopausal women, higher bone mineral density (BMD) was observed in the degenerated disk group at L5-S1, and in postmenopausal women, BMD was higher at specific lumbar and calcaneus sites associated with degenerated disks, highlighting an early-stage correlation between BMD and lumbar intervertebral disk degeneration [13]. Likewise, in a study conducted by Livshits et al [14], which involved a sample of 908 individuals, a significant positive correlation was observed between lumbar disc degeneration (LDD) and bone mineral density (BMD) in both the lumbar spine and hip regions. This correlation remained significant after adjusting for confounding factors. Pye et al. [9]. conducted a study involving a sample of 500 males and females, examining osteophytes, intervertebral disc narrowing, and endplate sclerosis in the lumbar spine. Their research revealed that, after adjusting for age, the bone mineral density (BMD) of all imaging features of intervertebral disc degeneration increased with higher grades. However, it’s important to note that their study established an association between BMD and intervertebral disc degeneration but did not establish a causal relationship. Regarding the biological mechanism, the study by Sun et al. suggested that during the treatment of osteoporosis, the calcification of the cartilaginous endplate hinders the delivery of nutrients and oxygen to the intervertebral disc, potentially resulting in disc degeneration [29]. Another study indicated that elevated bone mineral density (BMD) amplifies the static compressive force exerted on the endplates. This impedes the diffusion of essential nutrients to the disc level, thereby contributing to the development of intervertebral disc degeneration (IDD) [30].Furthermore, studies have found that in cases of lower vertebral bone density, there is an increase in the number of blood vessels, which improves blood supply to the cartilaginous endplate and slows down intervertebral disc degeneration [31]. These studies provide evidence for the association between bone mineral density and intervertebral disc degeneration (IDD). Nevertheless, it is essential to exercise caution when interpreting these findings, considering that they are all observational studies.

The relationship between bone mineral density and intervertebral disc degeneration remains a subject of significant debate. In a study conducted by Xiao ZF [32], a comparison was made between normal mice and those with induced osteoporosis resulting from uterine removal. The findings indicated disc degeneration in the ovariectomy group after 12 weeks. Additionally, Zhong et al.‘s study [33] delved into the influence of osteoporosis on endplate microarchitecture and vascularization in rhesus monkeys, employing micro-computerized tomography (micro-CT). Additionally, they conducted an in-depth analysis of the association between osteoporosis and IVD degeneration. Their findings revealed significant correlations, indicating that osteoporosis may contribute to endplate calcification and reduced vascularization, thereby exacerbating disc degeneration. In another study by Xiao Liang and colleagues [34], involving 324 patients, Hounsfield unit (HU) measurements in cervical vertebrae were examined in relation to intervertebral disc degeneration. The findings revealed that in both young and elderly patients, the CT Hounsfield Unit (HU) values consistently peaked in the upper section of the C4 vertebrae, gradually diminishing towards C3 and C7. Furthermore, as the severity of intervertebral disc degeneration increased, both upper and lower vertebral HU values showed a declining trend. These findings suggest that reduced bone mineral density (BMD) and vertebral osteoporosis may potentially trigger intervertebral disc degeneration.

Based on the aforementioned observational and animal studies, there is still considerable controversy surrounding the correlation and causation between bone mineral density and intervertebral disc degeneration, and no definitive conclusions have been reached. Additionally, the mechanisms of mutual influence between the two remain uncertain. While most clinicians believe osteoporosis may lead to disc degeneration among the elderly population, the reality often reveals concurrent presentations of both conditions in final diagnostic outcomes. Additionally, the commonly held notion that osteoporosis causes disc degeneration may be confounded by factors such as age. As individuals age, osteoporosis and disc degeneration frequently develop simultaneously. However, notably, higher bone density may potentially play a supportive role in the process of disc degeneration. Therefore, the temporal relationship between the development of these two diseases has remained unclear, and the presence of confounding factors such as diabetes, obesity, and age has complicated the assessment of their association.

We conducted a Mendelian randomization study to explore the causal relationship between osteoporosis and intervertebral disc degeneration. MR analysis utilized genetic variants as instrumental variables to make causal inferences regarding the impact of modifiable exposures on health- and disease-related outcomes, accounting for unobserved confounding variables [35]. Our study has shown a positive correlation between elevated levels of four types of bone mineral density and intervertebral disc degeneration. Our study indicates that, even after controlling for confounding factors related to disc degeneration—such as obesity, diabetes, hyperthyroidism and smoking—lumbar spine bone mineral density, heel bone mineral density, and total body bone mineral density continue to contribute to intervertebral disc degeneration. A recent observational study found that higher serum free thyroxine levels may also be associated with intervertebral disc degeneration. Therefore, we included hyperthyroidism in the multivariate analysis to reduce confounding factors [21].In clinical practice, emphasis should be placed on monitoring patients’ bone density status and considering its impact on intervertebral disc degeneration, followed by appropriate preventive measures. Engaging in moderate aerobic exercise and strength training is crucial for promoting skeletal health and circulation, facilitating blood vessel growth and nutrient supply. Regular bone density assessments and comprehensive health evaluations are recommended to promptly identify issues and take necessary actions. This was the first study to evaluate the causal association between BMD and IDD in both directions using an MR study design, combined with MVMR analysis and bidirectional MR analysis to improve the reliability of causal estimation. And the MR study design reduced the impact of confounding factors and reverse causation compared with previous observational studies. Finally, this study used a large sample size from the GWASs pooled data set and used SNPs that were strongly associated with exposure, so the estimates from this study are closer to facts. While we obtained positive results as mentioned above, our study still carries some limitations. Firstly, we did not control for the impact of age on intervertebral disc degeneration, given the known association between age, bone density, and disc degeneration. Additionally, intervertebral disc degeneration is influenced by numerous uncontrollable factors, each of which may introduce a certain degree of impact in MR studies. Lastly, the underlying mechanisms behind this association remain incompletely understood. Therefore, further comprehensive and in-depth research will be essential in the future to elucidate the mechanisms linking bone mineral density and intervertebral disc degeneration conclusively.

## Conclusions

In summary, this study represents the first-ever investigation utilizing both univariate and multivariate Mendelian Randomization (MR) approaches to explore the causal impact of bone mineral density (BMD) on intervertebral disc degeneration (IDD). Our findings reveal a significant positive causal relationship between total body bone mineral density (TB BMD), lumbar spine bone mineral density, femoral neck bone mineral density (FN BMD), and heel bone mineral density (eBMD) with IDD, thereby establishing higher bone density as a risk factor for intervertebral disc degeneration. Importantly, we did not observe any causal effect of IDD on these four measures of bone mineral density. Further research is needed to unravel the underlying mechanisms responsible for this causal relationship.

### Electronic supplementary material

Below is the link to the electronic supplementary material.

Supplementary Material 1

Supplementary Material 2

Supplementary Material 3

Supplementary Material 4

Supplementary Material 5

Supplementary Material 6

## Data Availability

Data used in the paper is free available in IEU GWAS database(https://gwas.mrcieu.ac.uk/datasets).

## Abbreviations

GWAS: Genome-wide association study
IV: Instrumental variable
IVW: Inverse variance weighted
MR: Mendelian randomization
OR: Odds ratio
SNP: Single nucleotide polymorphism. BMD = bone mineral density
IDD: Intervertebral disc degeneration
