# Supplementary figures and images for: Causal relationship between bone mineral density and intervertebral disc degeneration: a univariate and multivariable mendelian randomization study
Source: BMC Musculoskelet Disord. 2024 Jul 5;25:517. doi: 10.1186/s12891-024-07631-7 (PMC11225368; doi:10.1186/s12891-024-07631-7)

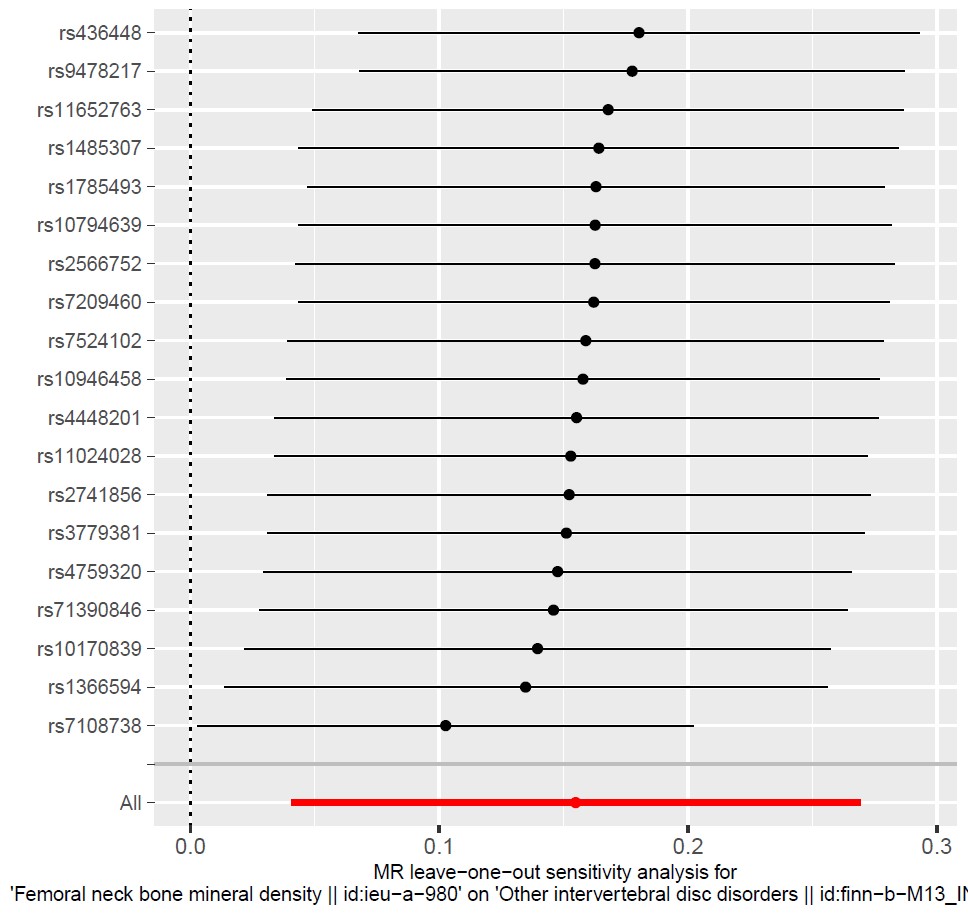


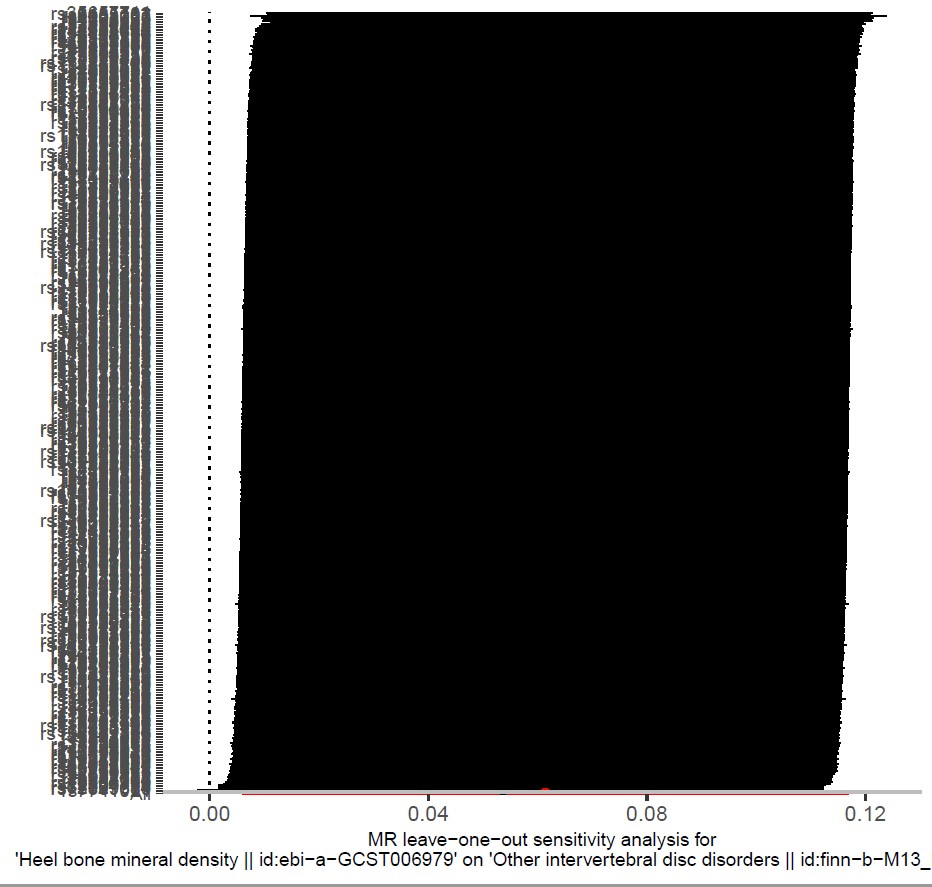


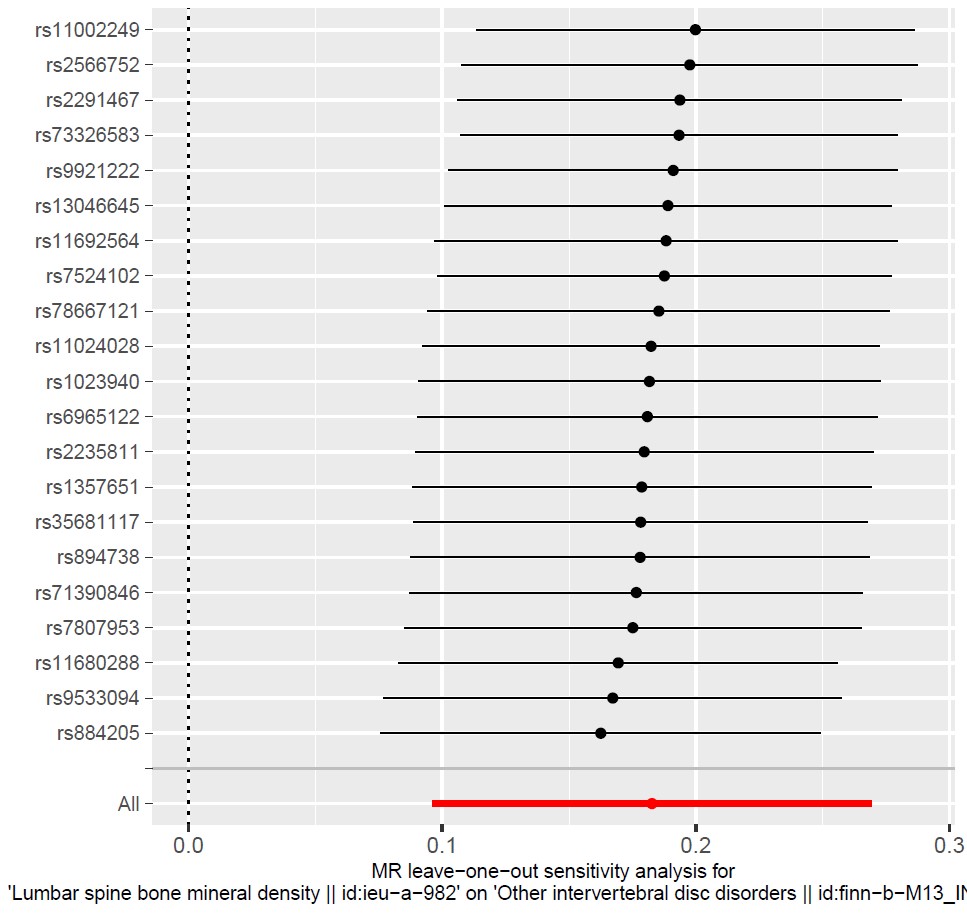


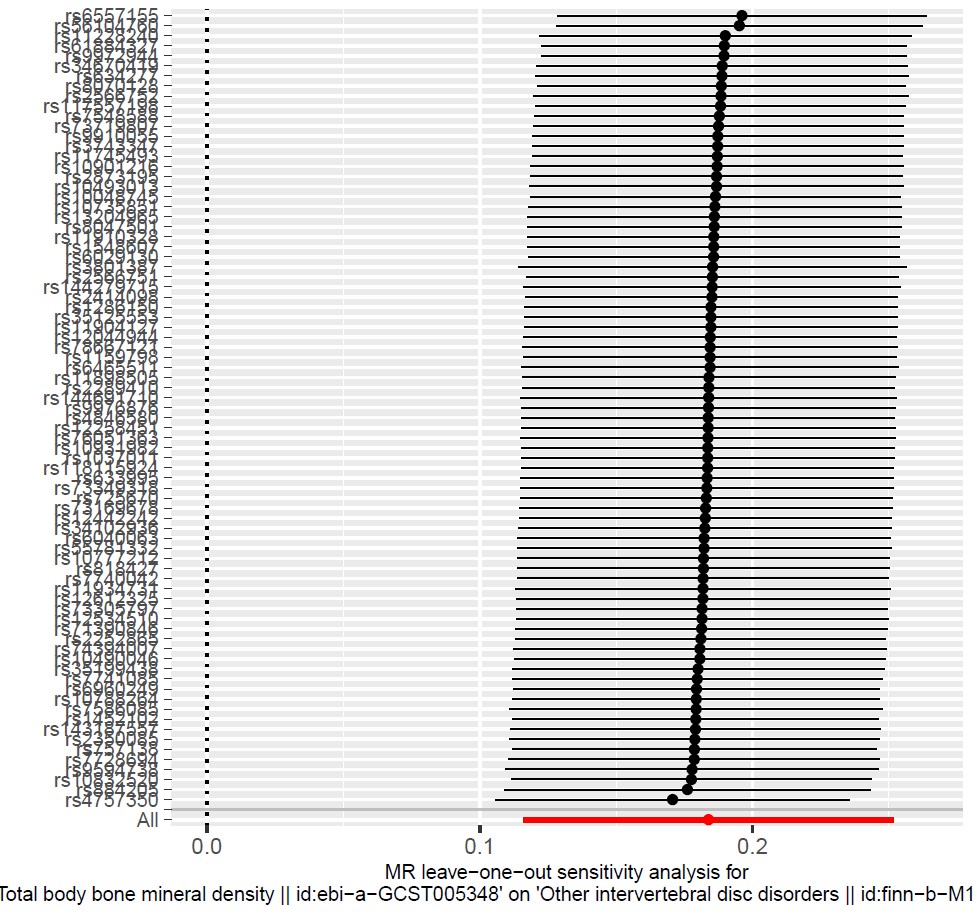

Supplement: Supplementary file 5 — Supplementary Material 5 [file 12891_2024_7631_MOESM5_ESM.docx]

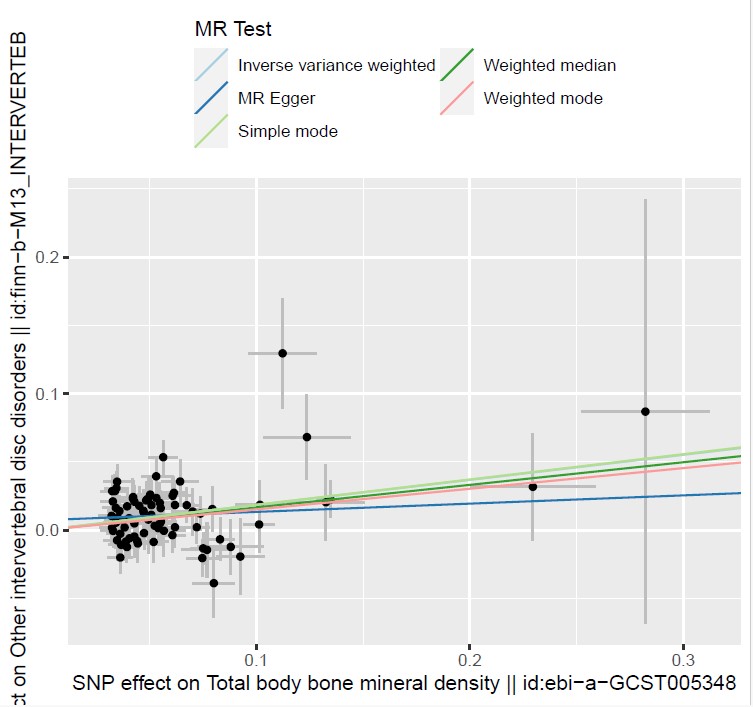

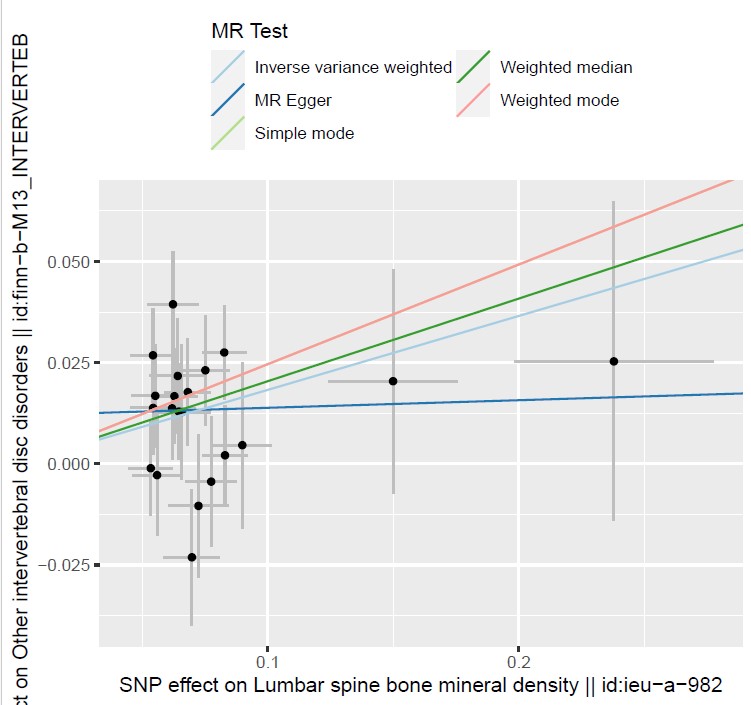

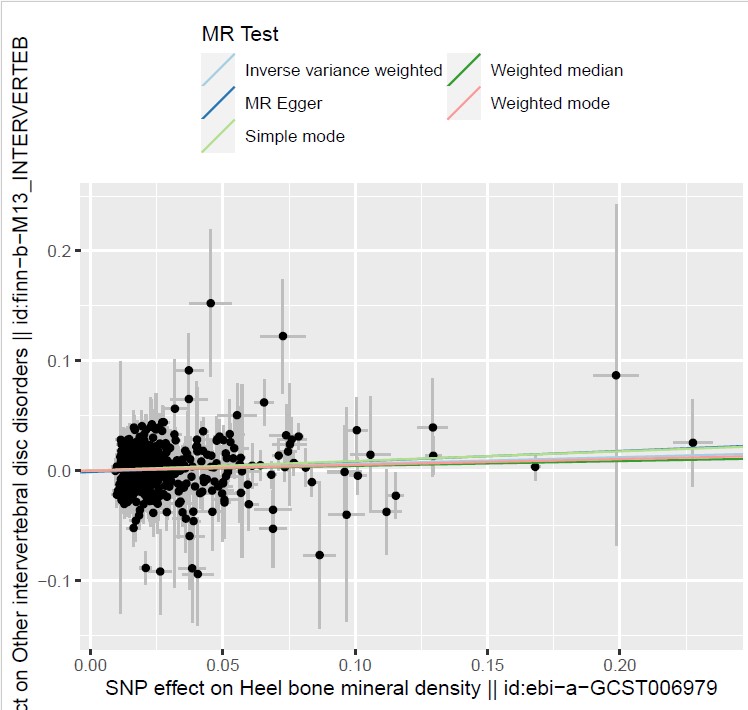

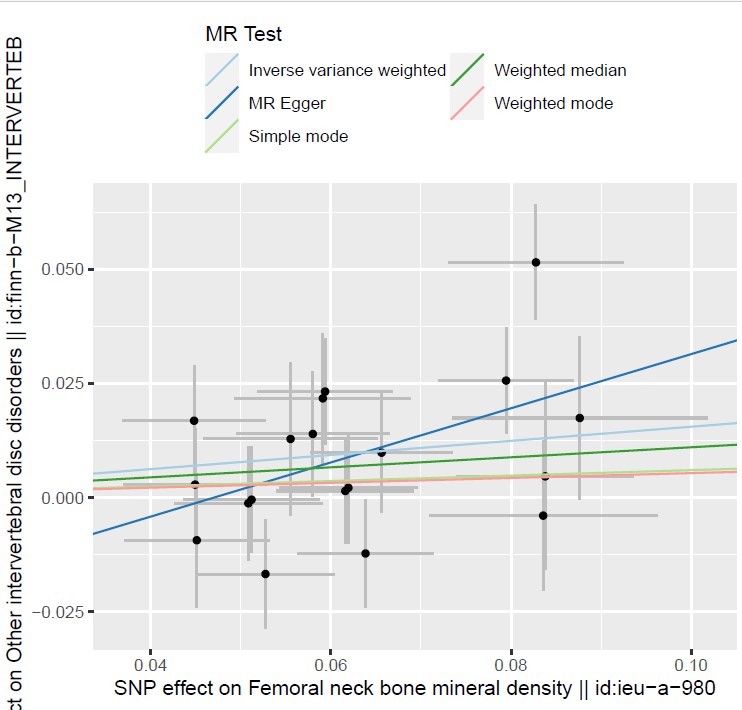

Supplement: Supplementary file 6 — Supplementary Material 6 [file 12891_2024_7631_MOESM6_ESM.docx]
